# Altered interoception in patients with borderline personality disorder: a study using heartbeat-evoked potentials

**DOI:** 10.1186/s40479-020-00139-1

**Published:** 2020-10-22

**Authors:** Vera Flasbeck, Stoyan Popkirov, Andreas Ebert, Martin Brüne

**Affiliations:** 1 LWL University Hospital, Department of Psychiatry, Psychotherapy and Preventive Medicine, Division of Social Neuropsychiatry and Evolutionary Medicine, Ruhr University Bochum, Alexandrinenstr. 1, D-44791 Bochum, Germany; 2 University Hospital Knappschaftskrankenhaus Bochum, Department of Neurology, Ruhr University Bochum, In der Schornau 23-25, 44892 Bochum, Germany

**Keywords:** Interoception, Heartbeat-evoked potentials, Autonomic nervous system, Borderline personality disorder, Alexithymia, Dissociation

## Abstract

**Background:**

Patients with borderline personality disorder (BPD) experience difficulties in emotional awareness (alexithymia), and often develop dissociative symptoms, which may reflect broader deficits in interoceptive awareness. Whether this is associated with alterations in cortical processing of interoception is currently unknown.

**Methods:**

We utilized an electrophysiological marker of interoception, i.e. heartbeat-evoked potentials (HEP), and examined its relationship with electrocardiographic correlates of autonomic nervous system (ANS) functioning (heart rate variability), and with self-report measures of alexithymia, dissociation and borderline symptom severity in patients with BPD.

**Results:**

Individuals with BPD had higher HEP amplitudes over frontal electrodes compared to healthy controls. Sympathetic ANS activity was greater in BPD patients than in controls. Across groups, HEP amplitudes were associated with parasympathetic activity over central electrodes and correlated with alexithymia over frontal electrodes.

**Conclusions:**

These findings support the idea that difficulties in emotional awareness in BPD are reflected in altered frontal electrophysiological markers of interception. Therefore, emotional awareness can be understood as failures of modulation between interoceptive and exteroceptive attention. Future research may aim to investigate whether altered interoception and its electrophysiological correlates are malleable by therapeutic intervention.

## Background

Borderline personality disorder (BPD) is characterized by self-injurious and risk-taking behavior, deficits in emotion regulation and poor impulse control, fragile self-images, unstable relationships and intensive fear of being abandoned [1]. Moreover, many patients with BPD have difficulties in identifying and describing their own feelings, referred to as alexithymia [2–5]. BPD is also frequently associated with trauma-related dissociative symptoms [6, 7]. Both, alexithymia and dissociation can be conceptualized as dysfunctional interoception [8]. Indeed, the perception of internal bodily signals and emotional awareness are physiologically closely related [9, 10], integrated in a conceptual framework referred to as the “Somatic Marker Hypothesis” (SMH [11];). In addition, there is also evidence for an association of dissociation and alexithymia in BPD [12, 13]. Interestingly, alexithymia in BPD does not seem to be associated with deficits of automatic visual processing of negative emotional stimuli, i.e. visual aspects of exteroception [14].

One promising approach to measure interoception has utilized heartbeat-evoked potentials (HEP) representing event-related potentials (ERPs) over scalp electrodes that are time-locked to the R-wave of the electrocardiogram (ECG). It has been proposed that interoceptive awareness correlates with the magnitude of HEP amplitudes [15–17]. Aside from intentional awareness, HEP waveforms were shown to be influenced by attentional and motivational factors [15–18], as assessed by heartbeat perception or resting state conditions. In general, the association of HEPs with awareness was reported to be more prominent over frontocentral electrodes compared to parietal brain regions [15, 18, 19] That is, cardiac afferent signals seem to be processed by frontocentral brain regions via feed-forward signals from the insular region, the anterior cingulate cortex and the somatosensory cortices [20–22]. In line with the SMH, interoception (and thus HEPs) has also been linked to emotional awareness [11, 23–27] and to autonomic nervous system (ANS) functioning as revealed by heartrate variability [28, 29].

Consistent with these theoretical considerations, altered HEPs have been found in psychopathological disorders that are clinically characterized by reduced capacity for interoception and poor awareness of own feelings. For instance, smaller HEP amplitudes have been observed in depression compared to controls [30], whereas increased amplitudes have been reported in social anxiety disorder [31, 32]. To date, only a few studies have investigated interoception in BPD. Behaviorally, Hart and colleagues (2013) did not find differences between patients with BPD and healthy control participants in a heartbeat detection task [33], whereas another study reported superior heartbeat detection in patients with personality disorders compared to patients with functional disorders and control participants [34]. As regards HEP, two studies found significantly reduced HEP amplitudes in patients with BPD compared to a healthy control group in a resting state condition [35, 36]. In particular, low HEP amplitudes were correlated with the severity of BPD symptoms, depressive symptoms, emotion dysregulation and emotional abuse during childhood [35, 36]. Similarly, another previous study investigates the impact of childhood trauma on interceptive accuracy and the associations with cortisol and heartrate in response to a socially evaluated cold pressure test in unaffected individuals. They reported that childhood trauma affected interoceptive accuracy after the stressor, with high levels of childhood trauma being related to increased difficulties in perceiving the heartbeat. However, interoceptive accuracy was unrelated to cortisol levels and heartrate. These findings seem to support the idea that chronic stress during childhood induces long-term changes of the stress system and finally leads to development of impaired functioning of neural circuits underlying successful brain–body communication [37]. Since childhood trauma is frequently reported by patients with BPD, the previous studies may suggest altogether that state specific, ANS functioning dependent cortical processing of bodily signals may be associated with core features of BPD. However, to the best of our knowledge, possible associations of HEP with alexithymia, dissociation and ANS functioning have not been studied in BPD.

Accordingly, we sought to examine the association of behavioral correlates of impaired interoception (i.e. alexithymia and dissociative symptoms) with physiological measures, including HEP and ANS markers in patients with BPD. As a secondary goal, we aimed to characterize the role of childhood traumatization in interoception. We hypothesized that alexithymia, dissociation and trauma would be associated with HEP amplitudes and that HEP was related to measures of both parasympathetic and sympathetic activity in BPD. As a hormonal marker of stress, we analyzed baseline cortisol levels, expecting that cortisol would be related to HEP amplitudes and ANS functioning.

## Methods

### Participants

Twenty patients diagnosed with BPD according to the criteria of the Diagnostic and Statistical Manual of Mental Disorders (DSM), 5th edition, were recruited from the LWL-University Hospital Bochum. The diagnoses were confirmed by SKID interviews (German version [38]). For comparison, 20 healthy controls (HC) were recruited via public advertising. One patient with BPD and one healthy control participant received an ACE inhibitor and one control participant received beta-blocker for hypertension treatment. These three participants were therefore excluded from the analysis. Only subjects aged 18 to 50 were included. The patients’ mean age was 31.2 years (*SD* = 10.4) and the mean age of the control group was 27.4 years (*SD* = 5.8). The groups did not differ in terms of age (*t* (28.1) = − 1.354, *p* = 0.180). In addition, there was no difference between groups in verbal intelligence (BPD: IQ mean: 98.6, *SD* = 16.4; HC: IQ mean 106.8, *SD* = 13.5; *t* (35) = 1.664, *p* = 0.105). In the patient group, 17 subjects were female. In the HC group, 17 females were included. Two participants were left-handed in the patients group and one left-handed control participants was included. All other subjects were right-handed. A history of psychiatric or neurological disorders in control participants were an exclusion criterion. Patients who had recent or regular benzodiazepine medication were excluded. For psychopharmacological medication and comorbid disorders in the patient group see Table 1. The prescribed antidepressants were mainly SSRIs, only three patients took additional tricyclic antidepressants and two patients took additional bupropion. Somatic disorders were asthma (*n* = 2 in BPD group), type-I diabetes (*n* = 2 in BPD group) and thyroid dysfunction corrected by L-thyroxin (*n* = 2 in BPD group, *n* = 1 in HC group). Participants with other severe somatic disorders were not included.

**Table 1:** Overview of medication and comorbid disorders in the group of patients with BPD

	N	%
**Comorbid disorders of patients with BPD**		
Depressive episode	12	63.2
Posttraumatic Stress Disorder	3	15.8
Anxiety/Phobic Disorder	1	5.3
Cannabis misuse	2	10.5
Alcohol misuse	2	10.5
Other substance misuse	1	5.3
**Medication**		
No regular medication	9	47.4
Antidepressant (mainly SSRI)	4	21.1
Antidepressant and antipsychotic drugs	3	15.8
Antiepileptic medication	2	10.5
Other (additional) psychoactive drugs	3	15.8

Note: N indicates the absolute number and % indicates the relative number of patients with the diagnosis or medication

### Questionnaires

Verbal IQ was measured using a multiple-choice vocabulary intelligence test (MWT-A [39];). Handedness was examined using the German version of the Edinburgh Handedness Scale [40]. Alexithymia was measured by the 20-item Toronto Alexithymia Scale (TAS-20 [41], German version [42]). Here, the means of three subscales “difficulty identifying feelings” (DIF), “difficulty describing feelings” (DDF) and “externally-oriented thinking” (EOT) and the total score (sum of all subscales) were calculated. The severity of dissociative symptoms was evaluated by the German version of the Dissociative Experiences Scale (DES; Fragebogen zu Dissoziativen Symptomen [43];). A mean score of all DES items was computed, as well as means of the questions belonging to the subscales “amnesia”, “depersonalization/derealization” and “absorption”. For assessing maltreatment and aversive experiences during childhood, the Childhood Trauma Questionnaire (CTQ [44, 45];) was used. We calculated subscales for “emotional abuse”, “physical abuse”, “sexual abuse”, “emotional neglect” and “physical neglect” by summing up the responses for the respective questions. The CTQ total score is the sum of all subscales. Finally, the short version of the Borderline-Symptom-List (BSL-23 [46]) was utilized to examine the severity of Borderline symptoms. The BSL-23 mean values are reported in the present study. Depressive symptoms were assessed by the Beck Depression Inventory II (BDI-II [47, 48];). For this questionnaires, the sum of all responses was computed resulting in one depression score.

### Salivary cortisol

In order to determine possible correlations between HEP, HRV and stress physiology, we collected saliva samples for the assessment of the cortisol levels during rest by using Salivette® collection devices (Sarstedt, Nuembrecht, Germany). Samples were collected directly after the EEG recording, in order to obtain cortisol levels during baseline. Until sample analysis, the Salivettes were stored at − 20 °C.

### Resting state EEG and ECG recording

The EEG was recorded from 32 passive Ag/AgCl scalp electrodes (10–20 system) by BrainVision Recorder (Version 1.20.001, Brain Products GmbH, Germany) with impedances kept below 5 kΩ, with a sampling rate of 250 Hz and a band-pass filter (0.3–70 Hz). For the ECG recording, two single-use silver and silver-chloride electrodes by Philips Medical Systems were placed on each forearm close to the wrist. The EEG and ECG signals were recorded while participants were seated in a dimly lit room for 8 min with eyes closed. The participants were asked two times to open their eyes for 10 s in order to avoid the subjects falling asleep.

### Analysis of heartbeat-evoked potentials (HEP)

For data analysis BrainVision Analyzer (Version 2.2.07383; Brain Products GmbH, Germany) was used. The whole recording session was analyzed and was visually inspected for muscle artifacts and re-referenced offline to the average mastoids. A 50-Hz-notch and band pass filters (0.1–35, 24 dB/octave roll-off Hz) were applied. Eye movements were corrected [49] and artifacts exceeding ±100 μV were excluded. EEG data was segmented according to the R-waves detected in the ECG signal (− 200 ms – 800 ms). Baseline correction for − 200 ms before the R- wave was conducted and finally, the HEP were averaged for each participant. For the whole sample, 533.4 (*SD* = 90.2) segments were averaged, with 553.6 (*SD* = 87.8) segments in the group of patients with BPD and 512.2 (*SD* = 90.2) segments in the HC group. Before averaging, 17.4 (*SD* = 16.6) segments were rejected due to artifacts with 21.5 (*SD* = 18.3) and 13.1 (*SD* = 13.8) segments for patients and controls, respectively. In accordance with previous studies (e.g. [35, 36]), we extracted the mean amplitudes in the timeframe of 455–595 ms after the R-wave for all scalp electrodes. HEP amplitudes of an additional timeframe from 250 to 450 ms after the R wave were exported according to previous work [21]. We also aimed to explore another timeframe ranging from 524 to 620 ms, based on a recent study reporting that this time window would specifically unveil differences between interoceptive and exteroceptive awareness [50]. HEP amplitudes of the frontal electrodes (F3, F4, F7, F8, Fz, Fp1, Fp2), central electrodes (C3, C4, Cz, FC1, FC2, CP1, CP2) and parietal/occipital electrodes (P3, P4, Pz, P7, P8, O1, O2) were averaged for further analyses.

In order to address the crucial point that confounding cardiac effects could contribute to HEP differences between the groups, we additionally analyzed the ECG mean amplitude in all selected timeframes.

### Analysis of cardiovascular data

For further analysis of the cardiovascular data, the filtered data was further processed with the software Kubios HRV Premium 3.0 by Kubios Oy (Version 3.1, Kuopio, Finland). We extracted the results of the mean heart rate (HR), the standard deviation of NN intervals (SDNN), the root mean square of the successive differences (RMSSD) and the LF/HF ratio (computed by Fast Fourier Transformation). With the software, we calculated a general stress index (SI), an index for the activation of the sympathetic autonomic nervous system (SNS index) and an index mirroring the activation of the parasympathetic ANS (PNS index). The SI calculation is based on the so-called mode amplitude (AMo = height of the normalised RR interval histogram), the most frequent RR-interval (Mo = median of the RR intervals) and MxDMn, which reflects the degree of RR interval variability (=difference between longest and shortest RR intervals). The SI index was calculated according to the following formula.

1. $$ SI=\kern0.5em \frac{AMo\ x\ 100\%}{2 Mo\ x\  MxDMn} $$

The PNS index is calculated from mean RR, RMSSD and high-frequency power (HF). The SNS index computation is based on the mean HR, the stress index SI and low-frequency power (LF). Both indices were computed as mean deviations from normal values [51]. Therefore, a PNS or SNS index value of zero indicates that the parameters reflecting parasympathetic or sympathetic activity are on average equal to the normal population average. Indices different from zero show how many standard deviations below or above the normal population average the parameter values are.

### Statistical analyses

All statistical analyses were performed using IBM SPSS Statistics for Windows, version 26 (IBM Corp., Armonk, NY). For comparisons of age and IQ between the two groups, independent t-tests were calculated and for comparisons of questionnaire data, nonparametric Mann-Whitney-*U*-Tests were used, because of deviations from normal distribution (normal distribution was checked by Shapiro-Wilk-Tests; whereby CTQ, BSL and DES scores deviated). The HEP amplitudes were analysed by a mixed-model ANOVA with the between subject factor *group* (BPD/HC) and within-subject factors *timeframe* (455–595 ms/ 250–450 ms/ 524–620 ms) and *scalp location* (frontal/central/parietal). Post-hoc analysis of significant effects was performed by using dependent and independent two-tailed t-tests. Greenhouse-Geisser corrected results are reported and as measures of effect size, partial η^2^ values and Cohen’s *d* for the ANOVA and t-tests are stated. Since ECG data was not normally distributed, Mann-Whitney-*U*-tests were used for comparisons of ECG amplitudes between groups in all three timeframes. In order to test whether the ECG amplitudes would predict HEP amplitudes, linear regression analyses were utilized. A MANOVA was calculated for the cardiovascular data and cortisol (as both are suggested to reflect ANS activity) with the factor *group* (BPD/HC). For the calculation of exploratory correlations between HEP amplitudes during the main timeframe from 455 to 595 ms and cardiovascular data and cortisol levels, Pearson correlation coefficients were computed for the whole sample and for the samples separated. We also performed a stepwise linear regression analysis including *group* and *PNS* index as predictor variables for *HEP over central electrodes* as the dependent variable. For correlations between HEP/cardiovascular data and questionnaires, Spearman correlations coefficients were used. We decided to focus on frontal and central electrodes, since HEP amplitudes are suggested to occur mainly over frontocentral brain areas. Bonferroni correction was applied due to multiple testing. For all other calculations, a significance level of *p* < 0.05 was chosen.

## Results

### Questionnaires

As expected, patients with BPD experienced significantly more dissociations and reported more severe BPD and depressive symptoms and alexithymia compared to the HC group (see Table 2). In addition, patients with BPD more often reported emotional and physical abuse and neglect during childhood.

**Table 2:** Psychometric properties of participants. Means, standard deviations (*SD*), medians and Mann-Whitney-U-Test statistics (*U, Z, p*) are reported. Significant differences between the groups are marked with*

Questionnaires & subscales	BPD			HC			Mann-Whitney U-test		
	Mean	*SD*	Median	Mean	*SD*	Median	*U*	*Z*	*p*
CTQ	66.7	23.3	66.0	31.3	4.9	30.0	39.0	−4.02	< 0.001*
Emotional abuse	17.9	6.6	19.0	6.1	1.8	5.0	22.0	−4.61	< 0.001*
Physical abuse	10.7	6.5	7.0	5.2	0.5	5.0	73.5	−3.40	0.002*
Sexual abuse	8.0	5.6	5.0	5.6	2.4	5.0	109.5	−2.48	0.061
Emotional neglect	18.9	6.3	21.0	7.4	2.8	6.5	30.0	−4.33	< 0.001*
Physical neglect	11.2	4.5	10.0	6.1	1.7	5.0	43.0	−4.01	< 0.001*
TAS-20	64.2	7.6	64.0	36.2	7.5	36.0	0.0	−5.20	< 0.001*
Difficulty Identifying feelings	25.2	3.6	25.0	11.3	4.6	11.0	0.0	−5.21	< 0.001*
Difficulty describing feelings	18.3	3.4	19.0	9.7	3.4	11.0	13.5	−4.80	< 0.001*
Externally-oriented thinking	20.7	3.5	20.0	15.2	4.0	16.0	45.5	−3.83	< 0.001*
Dissociative experiences scale	29.9	15.5	28.9	9.2	5.9	8.2	34.0	−4.16	< 0.001*
Amnesia	12.4	13.9	8.8	4.1	7.3	1.6	79.5	−2.80	0.004*
Depersonalization/derealization	31.8	20.9	35.0	2.5	3.3	0.8	39.0	−4.06	< 0.001*
Absorption	38.5	20.0	40.0	14.9	9.6	12.8	50.5	−3.66	< 0.001*
BDI-II	38.2	11.0	40.0	4.6	4.4	3.5	1.0	−5.17	< 0.001*
BSL-23	2.4	0.9	2.7	0.2	0.3	0.1	2.0	−5.41	< 0.001*

### Heartbeat-evoked potentials

**Fig. 1:**
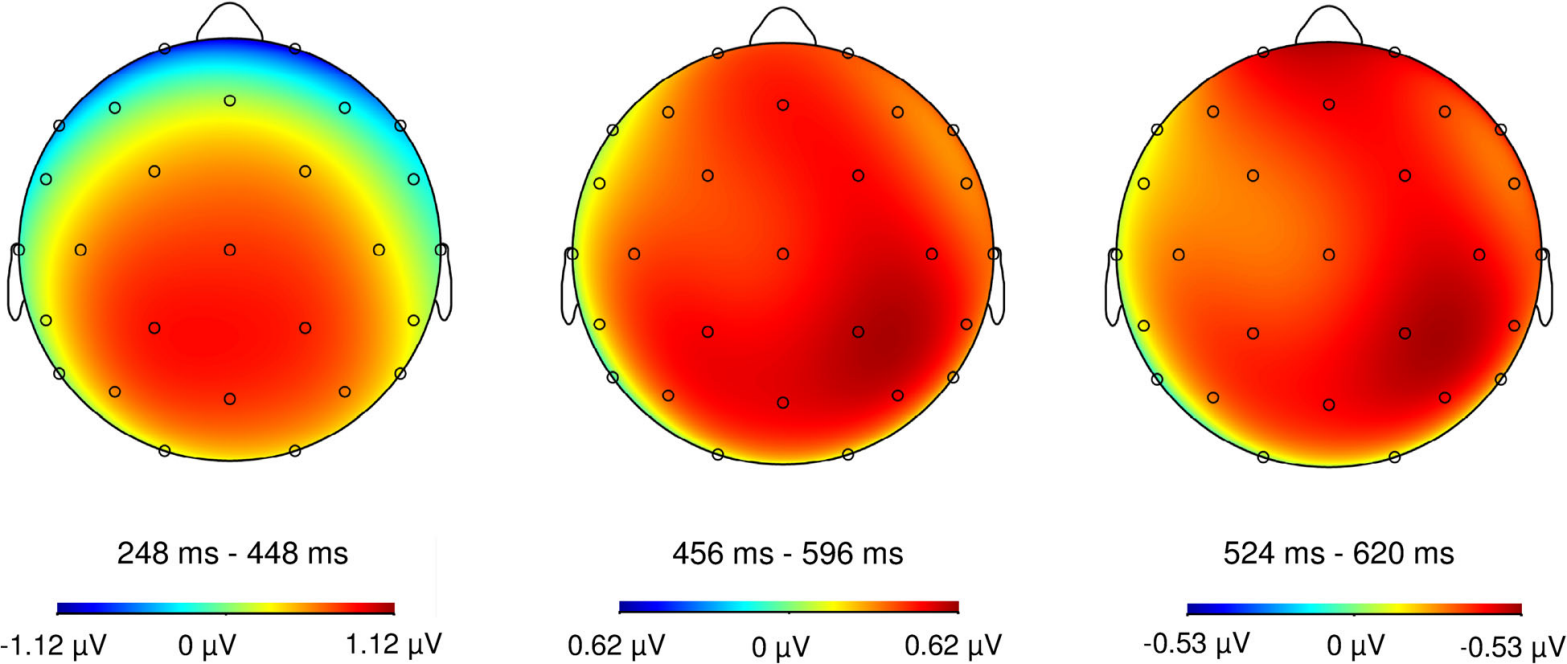
Topographic maps of heartbeat-evoked potentials in the three timeframes 248–448 ms, 456–596 ms and 524–620 ms after the R-wave. Data of healthy controls and patients with BPD are pooled

The ANOVA analysis with the factors *group, scalp location* and *timeframe* revealed significant main effects of *timeframe* (*F* (1.1, 38.0) = 8.81, *p* = 0.004; partial *η*^*2*^ = 0.201) and *scalp location* (*F* (1.7, 60.9) = 12.28, *p* < 0.001; partial *η*^*2*^ = 0.260). The main effect of *timeframe* indicates that the HEP amplitudes differed between the timeframes, whereas the amplitudes were maximal in the timeframe 455–595 ms (455–595 ms: *M* = 0.334, *SD* = 0.344; 250–450 ms: *M* = 0.179, *SD* = 0.440; 524–620 ms: *M* = 0.297, *SD* = 0.303; comparisons 455–595 ms vs. 250–450 ms: *t* (36) = 3.74, *p* = 0.001, *d* = 0.614; 455–595 ms vs. 524–620 ms: *t* (36) = 2.38, *p* = 0.023, *d* = 0.391; 250–450 ms vs. 524–620 ms: *t* (36) = 2.32, *p* = 0.026, *d* = 0.381). See Fig. 1 for topographic maps for all timeframes.

The main effect of scalp location shows that HEP amplitudes over central electrodes (*M* = 0.498, *SD* = 0.452) were higher compared to frontal (*M* = 0.090, *SD* = 0.547) and parietal electrodes (*M* = 0.222, *SD* = 0.359; comparisons central vs. frontal electrodes: *t* (36) = − 4.44, *p* < 0.001, *d* = − 0.730; central vs. parietal electrodes: *t* (36) = 4.09, *p* < 0.001, *d* = 0.672; frontal vs. parietal electrodes: *t* (36) = − 1.32, *p* = 0.196, *d* = − 0.216; see Fig. 1).

**Fig. 2:**
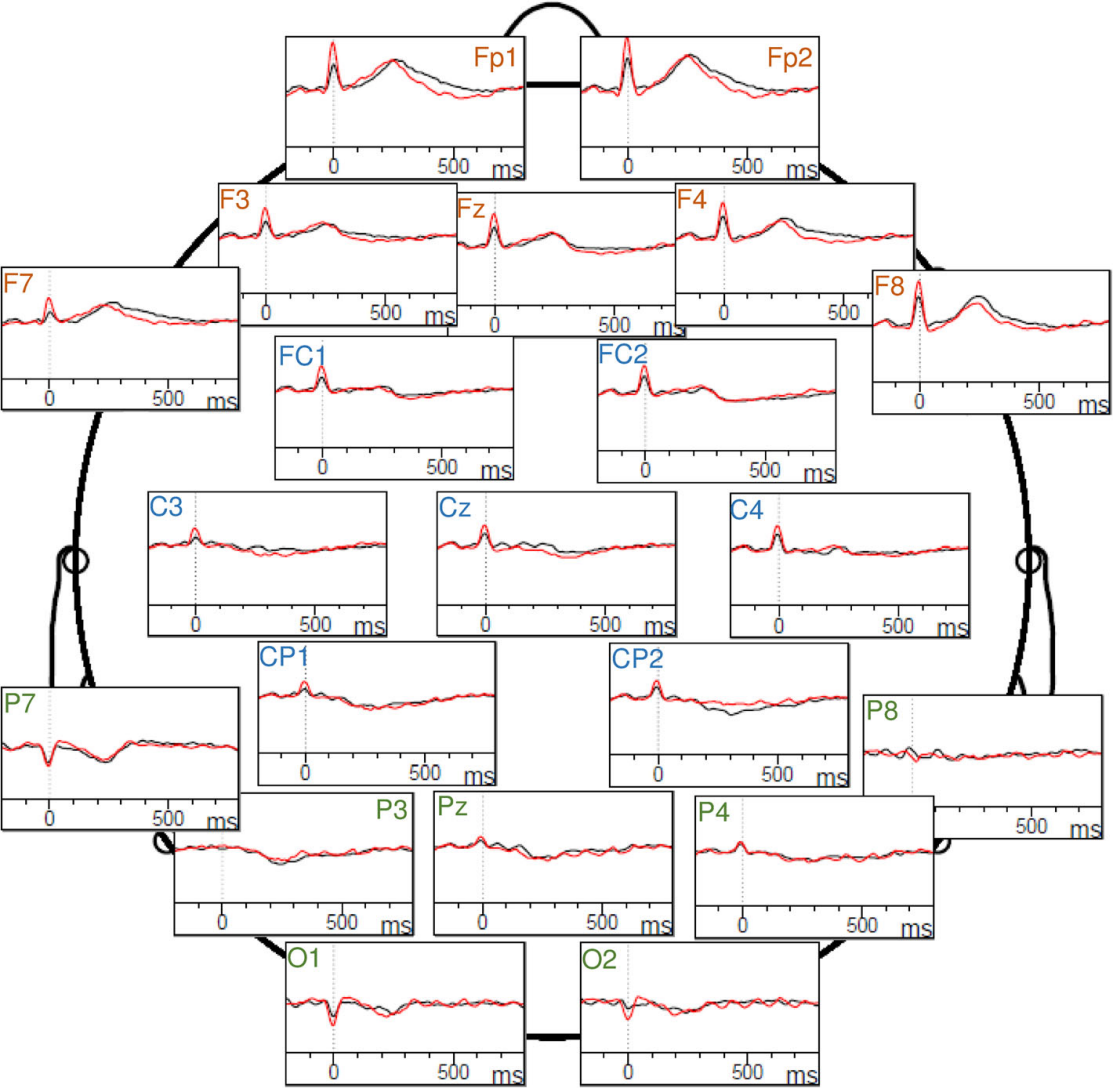
Grand-average waveforms of heartbeat-evoked potentials over the frontal electrodes (orange), central electrodes (blue) and parietal electrodes (green). Healthy controls are shown in black and patients with BPD are represented by red ERP lines

**Fig. 3:**
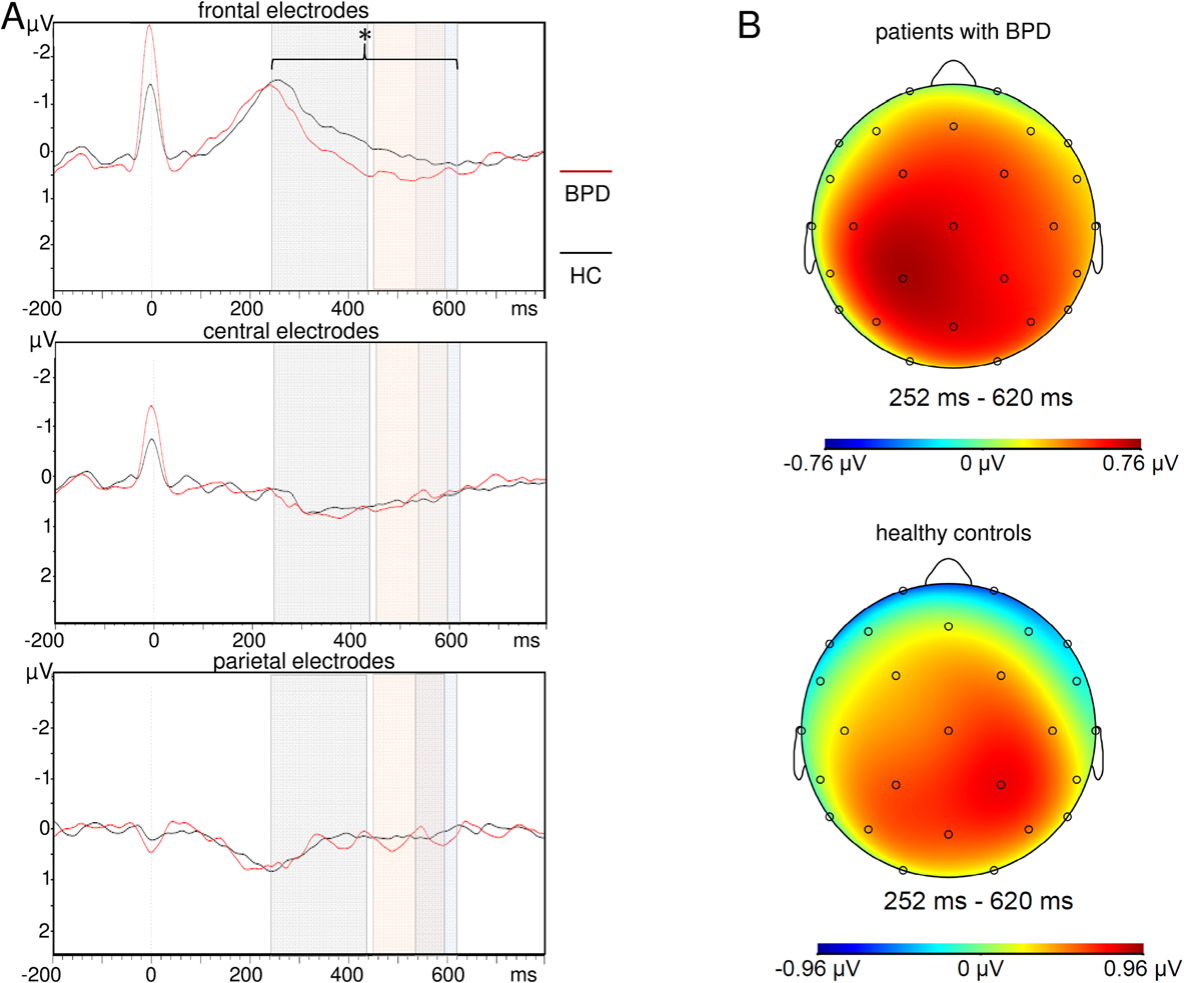
Pooled HEP waveforms for frontal, central and parietal electrodes for patients with BPD (red lines) and healthy control participants (black lines) (**a**). The selected timeframes are marked in A and the significant difference between groups is indicated by *. **b** shows the topographic maps of patients with BPD (upper map) and healthy controls (lower map) in the whole timeframe from 252 to 620 ms after the R-wave

Interestingly, a significant interaction of group with scalp location emerged (*F* (1.7) = 3.35, *p* = 0.048, partial *η*^*2*^ = 0.087). The interaction indicates that the HEP amplitudes were higher in patients with BPD compared to controls over frontal electrodes, whereas no differences occurred over central and parietal electrodes (frontal electrodes: BPD: *M* = 0.286, *SD* = 0.625; HC: *M* = − 0.117, *SD* = 0.362; *t* (35) = − 2.38, *p* = 0.023, *d* = − 0.391; central electrodes: BPD: *M* = 0.497, *SD* = 0.501, HC: *M* = 0.499, *SD* = 0.408; *t* (35) = 0.016, *p* = 0.987, *d* = − 0.003; parietal electrodes: BPD: *M* = 0.248, *SD* = 0.396; HC: *M* = 0.195, *SD* = 0.325; *t* (35) = − 0.44, *p* = 0.662, *d* = − 0.072; see Fig. 2 and Fig. 3a).

Further investigation of this interaction effect for the groups separately showed that the HEP amplitudes differed in controls between the scalp locations, with the maximal amplitude arising over central electrodes (central vs. frontal electrodes: *t* (17) = − 5.87, *p* < 0.001, *d* = − 1.382; central vs. parietal electrodes: *t* (17) = 3.01, *p* = 0.008, *d* = 0.709; frontal vs. parietal electrodes: *t* (17) = − 2.86, *p* = 0.011, *d* = − 0.673). In contrast, in the patients group, a difference was found only between central and parietal electrodes (central vs. frontal electrodes: *t* (18) = − 1.55, *p* = 0.140, *d* = − 0.355; central vs. parietal electrodes: *t* (18) = 2.70, *p* = 0.015, *d* = 0.619; frontal vs. parietal electrodes: *t* (18) = 0.24, *p* = 0.812, *d* = 0.055; see Fig. 3b).

Finally, an interaction of timeframe with scalp locations was found (*F* (1.7, 60.8) = 68.55, *p* < 0.001, partial *η*^*2*^ = 0.662). Post-hoc tests revealed that amplitudes were higher over central compared to parietal electrodes in the timeframe 455–595 ms in the whole group (central electrodes: *M* = 0.478, *SD* = 0.443; parietal electrodes *M:* = 0.194, *SD* = 0.352; frontal electrodes: *M:* = 0.331, *SD* = 0.533; central vs. parietal electrodes: *t* (36) = 4.10, *p* < 0.001, *d* = 0.673; frontal vs. central electrodes: *t* (36) = − 1.70, *p* = 0.097, *d* = − 0.280; frontal vs. parietal electrodes: *t* (36) = 1.52, *p* = 0.137, *d* = 0.250). In the timeframe of 524–620 ms, an additional difference was found between frontal and parietal electrodes (central electrodes: *M* = 0.385, *SD* = 0.388; parietal electrodes *M:* = 0.137, *SD* = 0.325; frontal electrodes: *M:* = 0.369, *SD* = 0.487; central vs. parietal electrodes: *t* (36) = 3.93, *p* < 0.001, *d* = 0.646; frontal vs. central electrodes: *t* (36) = − 0.21, *p* = 0.839, *d* = − 0.034; frontal vs. parietal electrodes: *t* (36) = 2.65, *p* = 0.012, *d* = 0.435), and in the early timeframe from 250 to 450 ms, all scalp locations differed from each other (central electrodes: *M* = 0.631, *SD* = 0.607; parietal electrodes *M:* = 0.336, *SD* = 0.466; frontal electrodes: *M:* = − 0.429, *SD* = 0.778; central vs. parietal electrodes: *t* (36) = 3.13, *p* = 0.003, *d* = 0.515; frontal vs. central electrodes: *t* (36) = − 0.812, *p* < 0.001, *d* = − 1.334; frontal vs. parietal electrodes: *t* (36) = − 4.995, *p* < 0.001, *d* = − 0.821, see Fig. 1).

### Cardiovascular measures

Differences between groups for heart rate-related measures and cortisol were calculated by a MANOVA. Here, a highly significant main effect of *group* emerged (*F* (8, 27) = 3.567, *p* = 0.006, partial *η*^*2*^ = 0.514). Post-hoc univariate ANOVAs for the dependent variables showed significant group differences for the SNS index, Stress index and SDNN, respectively. Thus, the activity of the sympathetic nervous systems, as measured by the SNS index, was higher in patients with BPD (see Table 3), as was the general Stress index. The SDNN was smaller in patients compared to controls. No significant effects were found for the parasympathetic measures, the PNS index and RMSSD. Heartrate differed between groups at trend level.

**Table 3:** Means, standard deviations (*SD*) and *F*-test statistics of cardiovascular measures and cortisol levels in patients with BPD and healthy participants. Significant differences between groups are marked with * and bold font

Variable	BPD		Controls		***F***-test statistics		
	Mean	***SD***	Mean	***SD***	***F***	***p***	partial ***η***^***2***^
PNS index	0.344	0.557	0.359	0.344	0.009	0.925	0.000
SNS index	**1.007**	**1.558**	**−0.038**	**1.261**	4.818	0.035*	0.124
Stress index	**13.603**	**5.916**	**8.924**	**3.695**	7.872	0.008*	0.188
Mean HR (bpm)	74.575	7.447	69.248	10.684	3.064	0.089	0.083
SDNN (ms)	**42.647**	**22.415**	**64.401**	**22.690**	8.354	0.007*	0.197
RMSSD (ms)	40.559	31.503	55.820	26.855	2.416	0.129	0.066
LF/HF ratio (FFT)	1.044	1.108	1.018	1.108	0.005	0.944	0.000
Cortisol	8.748	7.993	7.821	4.834	0.172	0.681	0.005

### Correlations

A significant correlation that survived correction for multiple testing emerged between HEP amplitudes over central electrodes and the PNS index. Moreover, symptom severity of BPD was related to the cardiovascular measures (Table 4).

**Table 4:** Correlations (*r* (*p*)) of heartrate-related measures with HEP (over frontal and central electrodes), cortisol levels and borderline symptom severity (BSL-23). Significant correlations are marked with * and printed in bold. These correlations survived Bonferroni correction (corrected for 6 factors, i.e. HEP frontal, HEP central, BSL-23, cortisol, heartrate and HRV-variables (whereby PNS index, SNS, Stress index, SDNN were pooled into a single one factor due to their close interrelation) resulting in *p* < 0.05/6 = 0.0083). Statistics of variables among each other and redundant results are not shown

	PNS index	SNS index	Stress index	SDNN	Mean HR	Cortisol	BSL-23
**HEP frontal**	0.3871 (0.018)	0.158 (0.351)	0.137 (0.420)	−0.277 (0.097)	0.116 (0.495)	−0.169 (0.325)	−0.248 (0.139)
**HEP central**	0.523 (0.001)*	−0.323 (0.051)	−0.2268 (0.108)	0.129 (0.446)	−0.391 (0.017)	− 0.399 (0.016)	−0.083 (0.626)
**BSL-23**	0.135 (0.425)	0.405 (0.013)	0.493 (0.002)*	−0.483 (0.002)*	0.362 (0.028)	−0.001 (0.994)	
**Cortisol**	−0.155 (0.365)	0.411 (0.013)	0.374 (0.025)	−0.163 (0.341)	0.336 (0.045)		

**Fig. 4:**
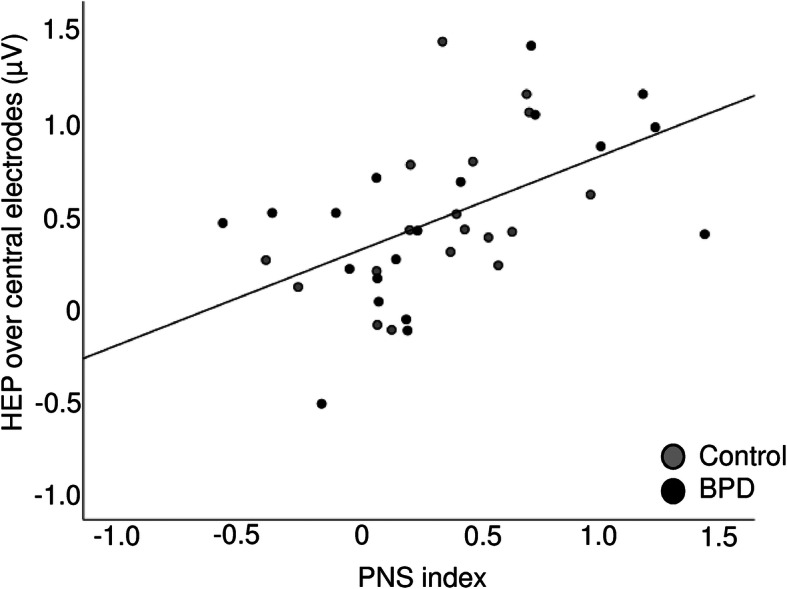
Scatter plot showing the association between HEP over central electrodes and the PNS index. Patients with BPD and healthy controls are represented by black and grey marks, respectively

A stepwise regression with *PNS index* and *group* as independent variables and *HEP over central electrodes* as the dependent variable revealed that only *PNS index* significantly predicted HEP magnitude (*F* (1, 36) = 13.158, *p* = 0.001; corrected *R*^*2*^ = 0.252; *b* = 0.523, *t* = 3.627, *p* = 0.001), whereas *group* was excluded from the equation (see also Fig. 4).

Additional correlations emerged between HEP and questionnaire data with high HEP amplitudes over frontal electrodes being associated with high alexithymia (difficulties identifying feelings score: *r* = 0.421, *p* = 0.010; whereby Bonferroni correction was applied for 4 factors: HEP frontal, HEP central, TAS score, and DES score, resulting in *p* < 0.05/4 = 0.013). No association was found for HEP over central electrodes, nor between HEP and dissociative symptoms.

Moreover, we investigated the correlations between the experiences of aversive events and traumatization during childhood and HEP amplitudes and stress measures (Table 5). Here, correlations reached significance only between CTQ scores and cardiovascular measures.

**Table 5:** Correlations (*r* (*p*)) between the items of the childhood trauma questionnaire and physiological data of HEP and cardiovascular measures. The significant correlations are marked with * and printed in bold. The correlations survived Bonferroni correction (corrected for 6 factors: HEP frontal, HEP central, CTQ score, cortisol, heartrate and HRV-variables (PNS index, SNS, Stress index, SDNN subsumed in a single one factor) resulting in *p* < 0.05/6 = 0.0083)

	Emotional abuse	Physical abuse	Sexual abuse	Emotional neglect	Physical neglect	Total score
**HEP frontal**	0.200 (0.236)	0.159 (0.348)	0.130 (0.443)	0.231 (0.170)	0.308 (0.064)	0.162 (0.338)
**HEP central**	0.141 (0.406)	0.034 (0.843)	−0.275 (0.099)	0.002 (0.991)	−0.062 (0.716)	− 0.032 (0.853)
**PNS index**	−0.066 (0.697)	0.025 (0.884)	−0.046 (0.789)	− 0.189 (0.263)	− 0.130 (0.442)	−0.152 (0.368)
**SNS index**	0.378 (0.021)	0.378 (0.021)	0.305 (0.065)	0.530 (0.001)*	0.343 (0.038)	0.475 (0.003)*
**Stress index**	0.479 (0.003)*	0.501 (0.002)*	0.362 (0.028)	0.617 (< 0.001)*	0.450 (0.005)*	0.563 (< 0.001)*
**SDNN**	−0.468 (0.003)*	− 0.383 (0.019)	− 0.290 (0.081)	−0.578 (< 0.001)*	− 0.419 (0.010)	−0.495 (0.002)*
**Mean HR**	0.313 (0.059)	0.286 (0.086)	0.342 (0.038)	0.509 (0.001)*	0.298 (0.074)	0.453 (0.005)*
**Cortisol**	−0.031 (0.856)	−0.027 (0.875)	0.072 (0.659)	0.028 (0.870)	−0.039 (0.823)	0.042 (0.808)

When correlations were analyzed for the groups separately, the correlations of HEP over central electrodes with PNS-index emerged in both groups, but would not survive correction for multiple testing. Since group differences occurred for psychometric questionnaires, no correlations were detected between HEP and TAS, DES and BSL-23 questionnaires within groups. In contrast, correlations between CTQ and cardiovascular measures, robust against Bonferroni correction, were found in the patients group (SNS index-CTQ-emotional neglect: *r* = 0.628, *p* = 0.004; SNS index-CTQ-total score *r* = 0.567, *p* = 0.011; stress index-CTQ-emotional neglect: *r* = 0.585, *p* = 0.009; stress index-CTQ-total score *r* = 0.571, *p* = 0.011; HR-CTQ-emotional abuse: *r* = 0.600, *p* = 0.007; HR-CTQ-emotional neglect: *r* = 0.714, *p* = 0.001; HR-CTQ-total score *r* = 0.635, *p* = 0.004).

### Analysis of confounding cardiac effects on HEP

Investigation of differences in ECG amplitudes in all timeframes between patients with BPD and healthy controls did not show any differences between groups (455–595 ms: *U* = 109.0, *Z* = − 1.88, *p* = 0.061; 250–450 ms: *U* = 119.0, *Z* = − 1.58, *p* = 0.118; 524–620 ms: *U* = 111.0, *Z* = − 1.82, *p* = 0.070). Similarly, linear regressions did not show a significant effect of ECG amplitudes on HEP amplitudes over frontal and central electrodes (455–595 ms: frontal electrodes *F* (1, 35) = 1.185, *p* = 0.284; central electrodes *F* (1, 35) = 0.801, *p* = 0.377; 250–450 ms: frontal electrodes *F* (1, 35) = 0.319, *p* = 0.576; central electrodes *F* (1, 35) = 1.556, *p* = 0.221; 524–620 ms: frontal electrodes *F* (1, 35) = 1.158, *p* = 0.289; central electrodes *F* (1, 35) = 0.660, *p* = 0.422).

## Discussion

The present study sought to explore the association of clinical correlates of poor interoceptive awareness (i.e. alexithymia and dissociation) in BPD with electrophysiological markers of interoception such as HEP. A second goal was to study correlations between HEP and stress-associated physiological measures. We found main effects of timeframe and scalp location, which indicate that HEP amplitudes were maximal over the selected timeframe from 455 to 595 ms and over central electrodes. No effect occurred for the timeframe of 524–620 ms, such that no conclusion can be made with regard to differences between intero- and exteroception in our sample. Importantly, the HEP findings could not be related to ECG characteristics, as shown by the additional analyses of ECG data.

Moreover, and in contrast to previous studies [35, 36], the HEP amplitudes were higher in patients with BPD compared to healthy participants, particularly over frontal electrodes. Differences between studies may originate from different methods and electrodes used. The studies [35, 36] analyzed HEP amplitudes of all recorded 60 scalp electrodes averaged. As it can be seen in Fig. 2, the polarity of HEPs varies with the electrode location. Thus, higher HEP amplitudes over frontal electrodes in the patients group is not in compelling opposition to previous findings. In contrast, overactivation of frontal brain regions could also negatively impact on interoception capacities. In fact, altered frontal activity was frequently reported in BPD (for review see [52]). In addition, in a previous study of our group, we found that altered frontal asymmetry was related to alexithymia in patients with BPD [3]. In line with these findings, HEP amplitudes over frontal electrodes correlated with alexithymia, but not with dissociative symptoms, the severity of borderline symptoms or childhood trauma. However, the correlation was significant only in the whole sample. In any event, the correlation between higher “difficulties identifying feelings” and higher HEP amplitudes seems to be contrary to previous research suggesting that alexithymia is associated with poor interoceptive awareness [25, 53–55]. This also raises the question whether high HEP amplitudes do indeed simply reflect high interoception? Because attention is also suggested to modulate HEP amplitudes [50], behavioral tasks requiring attention to the own heart per definition are difficult to compare to studies on HEPs during resting state conditions. Moreover, regarding alexithymia, or psychopathology in general, future research may clarify if associations between HEP and psychological characteristics depend on distinct brain regions. Schmitz and colleagues [36], as well as Müller et al. [35] reported inverse correlations between HEP amplitudes over all scalp electrodes averaged and childhood maltreatment, emotion dysregulation and borderline, depressive, dissociative and anxiety symptoms. However, Schulz and colleagues [56] did not find correlations between HEP amplitudes and alexithymia in a sample of patients with depersonalization/derealization disorder (DPD), nor did differences between healthy participants and subjects with DPD emerge. During a heartbeat perception task, HEP amplitudes were larger in healthy participants compared with resting state HEP amplitudes, whereas this difference was absent in the patient group. This could suggest difficulties of patients with DPD to focus their attention effectively on interoceptive signals [56]. Here, we could not confirm associations between HEP and borderline and dissociative symptoms, nor between childhood trauma and HEP amplitudes.

In line with predictions, another interesting finding was that the HEP amplitudes correlated with electrocardiographic measures of the arousal, such as parasympathetic activity. As expected, the cardiovascular measures differed between the BPD patients and controls, with patients showing greater SNS activity, higher stress indices, and smaller SDNN compared to controls. In addition, stress indices correlated with the current severity of the borderline disorder, assessed by the BSL-23 in the whole sample, as well as with a history of childhood trauma. These findings are in accordance with previous work demonstrating associations of childhood trauma with ANS functioning in BPD, as well as altered ANS functioning in this disorder [57–59]. These studies reported increased sympathetic activity and decreased parasympathetic activity in BPD compared to controls. A tentative explanation could be that childhood trauma is a major driving force for sympathetic hyperarousal as a function of threat detection, which in turn may affect the cortical representations of ANS in terms of altered HEP amplitudes. However, the nature of the present study precludes inference of causality, and the correlations of measures of childhood trauma with HEP failed to reach significance level. Another open question that could not be clarified by the present analysis is the role of stress hormones in interoception. Here, cortisol levels were neither related to high SNS activity, nor to HEP amplitudes. Similarly, Schulz and colleagues did not report a general effect of cortisol administration on HEP, but a divergent HEP amplitudes depending on eyes open and eyes closed conditions only after the administration of cortisol [60]. Regarding the ANS parameter heart rate variability, previous work proposed a connection between heart rate variability (HRV) and HEP in clinically unaffected participants [29], whereas such correlations were not found in individuals with DPD [56]. In our own study, the correlation between the PNS index and HEP amplitudes over central electrodes reached significance in the whole group, but would not survive correction for multiple testing if calculated separately for the two groups. An additional regression analysis revealed that parasympathetic activity was the only significant predictor for central HEP amplitude, whereas group was not. Therefore, more research is needed to clarify this possible relationship in larger samples, both clinical and non-clinical. Aside from differences in diagnostic features, it is also plausible to assume that divergent task instructions during HEP measurement played a role. In particular, HEP amplitudes seem to be larger under resting EEG conditions with eyes closed, compared to open eyes, which is intuitively understandable, as interoception can be sharpened by precluding input from other sensory systems [32, 50, 56, 60].

Conversely, heightened vigilance toward potential sources of threat in the external environment may come at the cost of reduced interoceptive awareness, clinically expressed by alexithymia and dissociative symptoms, decreased pain perception and general deficits in bodily self-awareness in BPD [8]. Low interoception in turn, may foster the development of unfavorable coping strategies, such as self-injurious behavior as a dysfunctional attempt to override interoceptive deficits by extremely strong sensory input, which frequently occurs in BPD. This idea is indirectly supported by research suggesting that interoception is neuroanatomically linked to the anterior insula region and anterior cingulate cortex (ACC) [21, 35, 61–63]. With regard to BPD, functional brain imaging suggests that altered activation of the insula and the ACC cortex is linked to altered emotion processing (e.g. [64–66]) and pain processing [65]. The preferential localization of HEP over frontocentral regions is in accordance with the involvement of the frontal cortex, the somatosensory cortex, ACC and insula in the cortical representation of cardiovascular signals [15, 18, 26, 35, 67]. Thus, the increased frontal HEP amplitudes could reflect heightened frontal activity, which might impair interoception.

It is important to note, though, that altered interoception is likely not specific for BPD. Instead, altered interoception may be involved in other psychiatric disorders, foremost trauma related disorders (e.g., post-traumatic stress disorder), but also affective disorders including depressive and anxiety disorders. Thus, transdiagnostic approaches may be fruitful to further explore the neural correlates of poor interoception.

### Limitations

The presented study has several limitations. First, since the sample comprised mainly female participants, the results are not generalizable for both sexes. Second, the sample size is limited, therefore replication of the findings in a larger sample is required. Third, the multiple correlation analyses were exploratory, bearing the risk of false positive findings. Fourth, around half of the patients included into the study received medication, which might modulate cardiac function and its cortical representation. Fifth, the present study lacked the examination of subjective interoception, such as heartbeat detection performance. Finally, according to a recent study, HEP may not only represent interoceptive awareness, but oscillations of interoceptive and exteroceptive signals [68], which could not be tested in the present study.

## Conclusion

This is the first study to show that HEP amplitudes over frontal electrodes differ between patients with BPD and healthy controls, and that the amplitudes of HEPs are associated with measures of alexithymia and PNS activity. Moreover, heart rate variability was related to borderline symptom severity and experiences of childhood trauma. Aside from the need to replicate these findings in larger samples, future research may also seek to explore if the electrophysiological correlates of altered interoception are malleable by therapeutic intervention, particularly psychotherapy.

## Data Availability

The data analyzed for the present study are available from the corresponding author on reasonable request.

## Abbreviations

ACC: Anterior cingulate cortex
BDI-II: Beck Depression Inventory II
BPD: Borderline Personality Disorder
BSL-23: Borderline-Symptom-List
CTQ: Childhood Trauma Questionnaire
DES: Dissociative Experiences Scale
DPD: Depersonalization/derealization disorder
DSM: Diagnostic and Statistical Manual of Mental Disorders
ECG: Electrocardiogram
EEG: Electroencephalography
ERP: Event-related potentials
HC: Healthy controls
HEP: Heartbeat-evoked potentials
HR: Heart rate
HRV: Heart rate variability
LF/HF ratio: Ratio between low and high frequency components of HRV spectra
PNS index: Index for the activation of the parasympathetic autonomic nervous system
RMSSD: Root mean square of the successive differences
SDNN: Standard deviation of NN intervals
SI: Stress index
SKID: Structured clinical interview (Strukturiertes klinisches Interview)
SMH: Somatic Marker Hypothesis
SNS index: Index for the activation of the sympathetic autonomic nervous system
TAS-20: Toronto Alexithymia Scale
